# Impact of tourniquet during total knee arthroplasty when tranexamic acid was used: a meta-analysis of randomized controlled trials

**DOI:** 10.1186/s13018-021-02898-1

**Published:** 2022-01-15

**Authors:** Changjiao Sun, Xiaofei Zhang, Qi Ma, Yan Tu, Xu Cai, Yonggang Zhou

**Affiliations:** 1 Department of Orthopedic, Beijing Tsinghua Changgung Hospital, School of Clinical Medicine, Tsinghua University, No. 168 Litang Road, Dongxiaokou Town, Changping District, Beijing, 102218 China; 2 Department of Clinical Epidemiology and Biostatistics, Beijing Tsinghua Changgung Hospital, School of Clinical Medicine, Tsinghua University, No.1 68 Litang Road, Dongxiaokou Town, Changping District, Beijing, 102218 China; 3 Department of Nursing, Beijing Tsinghua Changgung Hospital, School of Clinical Medicine,, Tsinghua University, No. 168 Litang Road, Dongxiaokou Town, Changping District, Beijing, 102218 China; 4 Department of Orthopaedic Surgery, The First Medical Centre, Chinese PLA General Hospital, No. 28 Fuxing Road, Beijing, 100853 China

**Keywords:** Tranexamic acid, Blood loss, Total knee arthroplasty, Tourniquet, Randomized controlled trials

## Abstract

**Introduction:**

The efficacy of tourniquet use during primary total knee arthroplasty (TKA) is thought to reduce intraoperative blood loss, improve surgical exposure, and optimize cement fixation. Tranexamic acid (TXA) use can decrease postsurgical blood loss and transfusion requirements. This review aimed to appraise the effects of tourniquet use in TKA for patients with tranexamic acid use.

**Methods:**

A meta-analysis was conducted to identify relevant randomized controlled trials involving TXA plus a tourniquet (TXA-T group) and use of TXA plus no tourniquet (TXA-NT group) in TKA. Web of Science, PubMed, Embase, Cochrane Controlled Trials Register, Cochrane Library, Highwire, CNKI, and Wanfang database were searched from 2010 through October 2021.

**Results:**

We identified 1720 TKAs (1690 patients) assessed in 14 randomized controlled trials. Compared with the TXA-NT group, the TXA-T group resulted in less intra-operative blood loss (*P* < 0.00001) and decreased duration of surgery (*P* < 0.00001), however more hidden blood loss (*P* = 0.0004) and less knee range of motion (*P* < 0.00001). No significant differences were found between two groups in terms of decrease in hemoglobin (*P* = 0.84), total blood loss (*P* = 0.79), transfusion rate (*P* = 0.18), drainage volume (*P* = 0.06), Visual Analogue Scale (VAS) at either the day of surgery (*P* = 0.2), 1 day (*P* = 0.25), 2 day (*P* = 0.39), 3 day (*P* = 0.21), 5 day (*P* = 0.21), 7 day (*P* = 0.06) or 1 month after surgery (*P* = 0.16), Hospital for Special Surgery (HSS) score at either 7 day (*P* = 0.10), 1 month (*P* = 0.08), 3 month (*P* = 0.22) or 6 month after the surgery (*P* = 0.92), Knee circumference (*P* = 0.28), length of hospital (*P* = 0.12), and complications such as intramuscular venous thrombosis (*P* = 0.81), deep venous thrombosis (*P* = 0.10), superficial infection (*P* = 0.45), deep wound infection (*P* = 0.64), and delayed wound healing (*P* = 0.65).

**Conclusion:**

No big differences could be found by using or not tourniquet when use the TXA, though some benefits are related to operation time and less intra-operative blood loss by using tourniquet and TXA, Using the tourniquet was related to more hidden blood loss and less knee range of motion. More adequately powered and better-designed randomized controlled trials (RCTs) studies with long-term follow-up are required to validate this study.

## Introduction

Tourniquet use has been considered an essential element of the total knee arthroplasty (TKA). Many surgeons apply a tourniquet during TKA to reduce blood loss and operative times, improve surgical exposure, optimize cement fixation, and increase tissue concentrations of antibiotic drugs through intraosseous regional administration [1–5]. However, the once highly regarded advantages of tourniquet use have come under great scrutiny in light of its potential disadvantages. Issues which bring its use into question included reperfusion injury [6], patellar tracking issues [7], increased perioperative pain [8, 9], increased postoperative limb swelling [10, 11], decreased postoperative range of motion (ROM) [12], delayed rehabilitation [12], increased risk of thrombosis [13, 14], more frequent wound complications [15–17], and its negative effect on patients with vascular disease [18]. More recently, as a new strategy for reducing blood loss, perioperative administration of tranexamic acid (TXA) has gained popularity during TKA, mitigating some of the adverse effects of tourniquet use. Several studies have confirmed that TXA significantly reduces blood loss and transfusion requirements without increasing venous thrombotic events [19–21]. Although there are many systematic reviews and meta-analysis comparing tourniquet use and non-tourniquet use during TKA, there was no meta-analysis comparing the effects of TXA plus a tourniquet and the use of TXA plus no tourniquet. Therefore, we compare the impact of TXA plus a tourniquet and use of TXA plus no tourniquet in TKA. This review aimed to appraise the effects of tourniquet use in TKA for patients with tranexamic acid use.

## Methods

### Protocol and registration

The study protocol was registered with International prospective register of systematic reviews (PROSPERO), and the registration number was CRD42020185403. This meta-analysis was performed using a predetermined protocol following the Preferred Reporting Items for Systematic Reviews and Meta-Analyses (PRISMA) statement to assess the results' quality to make sure our meta-analysis's results reliable and veritable.

### Search strategy

A meta-analysis was conducted to identify relevant randomized controlled trials involving TXA plus a tourniquet (TXA-T group) and use of TXA plus no tourniquet (TXA-NT group in TKA. Web of Science, PubMed, Embase, Cochrane Controlled Trials Register,, Cochrane Library, Highwire, CNKI, and Wanfang database were searched from 2010 through October 2021. The keywords used were “total knee replacement,” “total knee arthroplasty,” “tourniquet,” “tranexamic acid,” “TXA,” “randomized controlled trials” in conjunction with Boolean operators “AND” or “OR.” We used Review Manager Software for MAC to perform the meta-analysis.

### Inclusion criteria

Studies were eligible if (1) the intervention was patients undergoing primary TKA using TXA and a tourniquet (TXA-T group); (2) the comparator was patients undergoing primary TKA using TXA and without tourniquet use (TXA-NT group); (3) the design of the study was a randomized controlled trial (RCTs); (4) the clinical outcome data were intra-operative blood loss (IBL), hidden blood loss (HBL), total blood loss (TBL), drainage volume, decrease in hemoglobin level, transfusion rate, Visual Analogue Scale (VAS) score, Hospital for Special Surgery (HSS) score, knee circumference, knee range of motion (ROM), length of stay (LOH), complications including intramuscular venous thrombosis (IMVT), deep venous thrombosis (DVT), superficial infection, deep wound infection, delayed wound healing. (5) The studies were required to contain at least one clinical outcome data; the exclusion criteria were as follows: (1) observational studies; (2) non-RCTs; (3) studies with insufficient clinical outcome data.

### Data extraction process

Two reviewers (C.J.S and Q.M.) used a standardized form to extract data. A third reviewer (X.C) was used to resolve disagreements in eligibility, data extraction, or quality assessment. Extracted data included the primary data based on the following: first author, year of publication, participants, age, gender, body mass index, diagnosis, anesthesia, prothesis, patellar resurfacing, tourniquet pressure, tourniquet realizing time, TXA administration, drainage, thromboprophylaxis.

### Assessment of studies

The studies' methodological quality was assessed following the instructions in the Cochrane Handbook for Systematic Reviews of Interventions.

### Statistical Analysis

RevMan software (version 5.4; The Cochrane Collaboration) was used for the analysis. The statistical heterogeneity was tested with the *X*^2^ test and *I*^2^ test. *I*^2^ < 25% was considered low statistical heterogeneity, *I*^2^ < 50% moderate statistical heterogeneity, and *I*^2^ < 75% high statistical heterogeneity. If the *P* value of heterogeneity was less than 0.1, heterogeneity would exist. Then, the random-effects model was used for meta-analysis. Data were summarized as the ratio of relative risk (transfusion rate, complications including the rate of IMVT, DVT, superficial infection, deep wound infection, delayed wound healing.) or the difference between means (IBL, HBL, TBL, drainage volume, decrease in hemoglobin level, VAS score, HSS score, knee circumference, knee ROM and LOH). For studies that did not report standard deviations (SDs), it was calculated from *P* values, confidence intervals, or standard errors. The results were considered as a statistically significant difference when *P* values were less than 0.05.

## Results

**Fig. 1:**
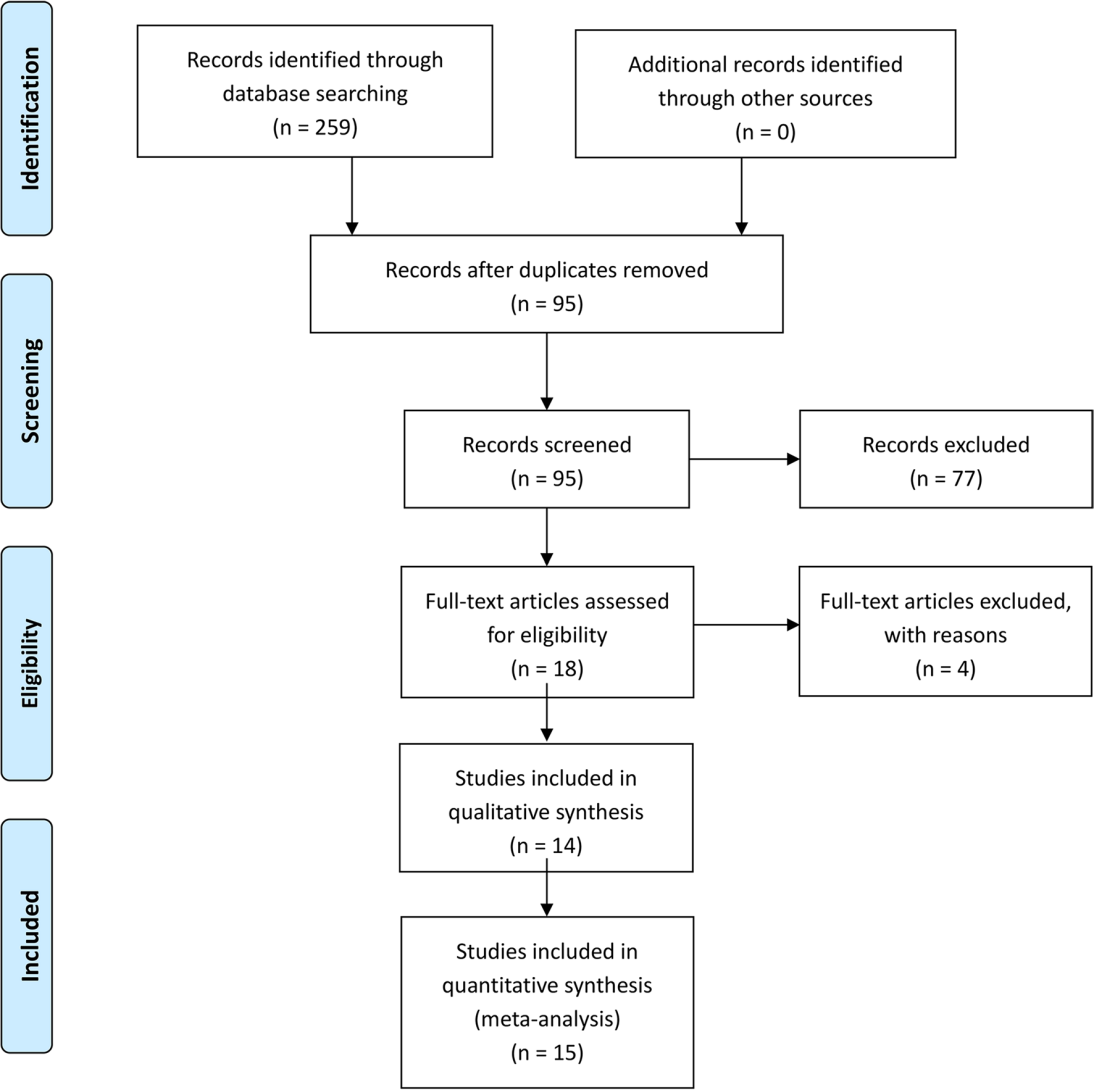
The search results and selection procedure. The literature search identified 259 citations. Of these, 164 duplicates were removed. After examining the titles and abstracts of the 95 remaining articles, we excluded 77 papers according to the inclusion and exclusion criteria; the full text of 18 articles was retrieved. Fourteen articles were assessed for eligibility. In Palanne’s article, there were two subgroups comparing TXA + tourniquet group with TXA + NT group. So we divided the study into two groups. Finally, we identified 1720 TKAs (1690 patients) assessed in 15 randomized controlled trial

The search strategy identified 259 studies, of which 245 were excluded after screening in Fig. 1. The literature search identified 259 citations. Of these, 164 duplicates were removed. After examining the titles and abstracts of the 95 remaining articles, we excluded 77 papers according to the inclusion and exclusion criteria; the full text of 18 articles was retrieved. Because we could not acquire sufficient data in one article, and four studies were non-RCTs. Hence, four studies were excluded. Fourteen articles were assessed for eligibility. In Palanne’s [22] article, there were two subgroups comparing TXA + tourniquet group with TXA + NT group. One is the spinal anesthesia subgroup, The other is the general anesthesia subgroup. So we divided the study into two groups: Palanne 2020 (1) and Palanne 2020 (2). Finally, we identified 1720 TKAs (1690 patients) assessed in 15 randomized controlled trials [2, 22–34]. Study baseline characteristics and general intervention information are summarized in Tables 1, 2, 3 and 4.

**Table 1:** The detailed baseline characteristics information

The detailed baseline characteristics information						
Author/year	Patients	Knees	Mean age (years)	Female gender (%)	BMI	Diagnosis
*Tourniquet use with TXA/No tourniquet use with TXA*						
Alexandersson 2018	38/43	38/43	68/69.7	52.6/48.8	28.6/27.9	38OA/43OA
Concina 2019	50/50	50/50	NA	NA	NA	NA
Ejaz 2014	33/31	33/31	68/68	45.5/45.2	25/25	33OA/31OA
Huang 2017	50/50	50/50	66.2/65.1	64/68	25.1/24.4	50OA/50OA
Ma 2017	31/32	31/32	66.8/67.2	61.3/65.6	24.38/24.02	31OA/32OA
Palanne 2020^1^	101/99	101/99	64/63	72.3/58.6	30.7/30.8	101OA/99OA
Palanne 2020^2^	99/96	99/96	63/65	62.6/61.5	30.5/29	99A/96OA
Wang 2017	30	30/30	65.9/65.9	86.7/86.7	26.6/26.6	30OA/30OA
Wang 2019	30/30	30/30	62.8/64.1	90/73.3	23.48/23.68	30OA/30OA
Xie 2017	45/45	45/45	66.2/66.1	85/75	26.1/25.9	NA
Xue 2018	30/30	30/30	68.2/69.1	60/53.3	NA	30OA/30OA
Yu 2017	40/40	40/40	60.65/62.6	NA	NA	40OA/40OA
Zak 2021	161/166	161/166	66.5/67.6	57/66	30.55/30.63	161OA/166OA
Zeng 2021	50/50	50/50	68.44/68	84/86	25.34/26.13	50OA/50OA
Zhou 2017	72/68	72/68	66.8/69.1	81.9/89.7	26.1/25.7	50OA/52OA; 22RA/16RA

The detailed baseline characteristics information, including the number of TKAs, age, gender, BMI, and two groups' diagnosis

OA, osteoarthritis; RA, rheumatoid arthritis; BMI, body mass index; TXA, tranexamic acid

**Table 2:** The detailed information about surgery

The detailed information of surgery				
Author/year	Anesthesia	Prothesis	Patellar resurfacing	Drainage
Alexandersson 2018	Spinal/general anesthesia,	NexGen fixed bearing (Zimmer)	No	No
Concina 2019	NA	Triathlon® (Stryker) and Attune® (DePuy)	NA	No
Ejaz 2014	Spinal anesthesia	NexGen fixed bearing (Zimmer)	Yes	No
Huang 2017	General anesthesia	NA	NA	Yes
Ma 2017	General anesthesia and FNB	PS, PFC (DePuy)	NA	Yes
Palanne 2020^1^	Spinal anesthesia	Triathlon® (Stryker)	Yes	No
Palanne 2020^2^	General anesthesia	Triathlon® (Stryker)	Yes	No
Wang 2017	General anesthesia	GenesisII (Smith&Nephew) or NexGen (Zimmer)	No	Yes
Wang 2019	General anesthesia	PS Haixing (Weihai)	NA	Yes
Xie 2017	General anesthesia	PS (DePuy)	No	Yes
Xue 2018	General anesthesia	NA	No	Yes
Yu 2017	Spinal anesthesia	NA	Yes	Yes
Zak 2021	NA	NA	NA	No
Zeng 2021		PS, PFC (DePuy)	NA	Yes
Zhou 2017	General anesthesia	PS, PFC (DePuy)	NA	Yes

The detailed information of surgery including anesthesia, prosthesis, patellar resurfacing, and drainage of two groups

FNB, femoral nerve block; PS, posterior cruciate-stabilizing; CR, cruciate retaining

**Table 3:** The detailed information of tourniquet use

Author/year	Tourniquet pressure	Tourniquet realizing Time	TXA administration
Alexandersson 2018	300 mmHg	After bandage applied	Intravenously, 1 g, 10 min before surgery
Concina 2019	300 mmHg	Before wound closure	Intravenously, 15 mg/kg, 20 min before surgery and after 4 h
Ejaz 2014	250 mmHg	After bandages applied	Orally, 1 g, before surgery; orally, 0.5 g 3 h after surgery
Huang 2017	100 mm Hg above systolic pressure	NA	Intravenously, 20 mg/kg, 5 to 10 min before the skin incision; Intravenous, 10 mg/kg, 3, 6, 12, and 24 h after operation; Topical, 1 g, intraoperatively
Ma 2017	100 mm Hg above systolic pressure	NA	Intravenously, 20 mg/kg, anesthesia induction; Topical, 1 g, intraoperatively; Intravenous, 10 mg/kg, 3, 6, 12, 24 h after anesthesia induction
Palanne 2020^1^	250 mmHg	After bandages applied	Intravenously, 1 g, 5 min before surgery; Topical, 1 g, intraoperatively; 1 g, 3 h, 6 h after surgery
Palanne 2020^2^	250 mmHg	After bandages applied	Intravenously, 1 g, 5 min before surgery; Topical, 1 g, intraoperatively; 1 g, 3 h, 6 h after surgery
Wang 2017	300 mmHg	After bandages applied	Intravenously, 1 g, 15 min before surgery; Topical, 1 g, intraoperatively
Wang 2019	NA	After bandages applied	Intravenously, 1 g, 15 min before surgery; Topical, 1 g, intraoperatively; Intravenously, 1 g, 3 h after surgery
Xie 2017	100 mm Hg above systolic pressure	After bandages applied	Intravenously, 20 mg/kg, 10 min before surgery; Topical, 60 ml, intraoperatively
Xue 2018	100 mm Hg above systolic pressure	After fascia layer closed	Intravenously, 1 kg, 30 min before surgery
Yu 2017	300 mmHg	After bandages applied	Topical, 1 g, intraoperatively
Zak 2021	NA	NA	Intravenously, two dose of 1 g, before surgery and during wound closure
Zeng 2021	100 mmHg above systolic blood pressure	After bandages applied	Intravenously, 1 kg, before surgery
Zhou 2017	NA	NA	Intravenously, 1 g, at the initiation of the surgery and just before closure

The detailed information of tourniquet pressure, tourniquet inflation time, tourniquet realizing time of two groups

**Table 4:** The detailed information of TXA and Thromboprophylaxis drugs

Author/year	TXA administration	Thromboprophylaxis drugs
Alexandersson 2018	Intravenously, 1 g, 10 min before surgery	Low-molecular weight heparin
Concina 2019	Intravenously, 15 mg/kg, 20 min before surgery and after 4 h	Enoxaparine 4000 IU
Ejaz 2014	Orally, 1 g, before surgery; orally, 0.5 g 3 h after surgery	Rivaroxaban (10 mg/day)
Huang 2017	Intravenously, 20 mg/kg, 5 to 10 min before the skin incision; Intravenous, 10 mg/kg, 3, 6, 12, and 24 h after operation; Topical, 1 g, intraoperatively	Enoxaparine 4000 IU
Ma 2017	Intravenously, 20 mg/kg, anesthesia induction; Topical, 1 g, intraoperatively; Intravenous, 10 mg/kg, 3, 6, 12, 24 h after anesthesia induction	Enoxaparine 4000 IU
Palanne 2020^1^	Intravenously, 1 g, 5 min before surgery; Topical, 1 g, intraoperatively; 1 g, 3 h, 6 h after surgery	NA
Palanne 2020^2^	Intravenously, 1 g, 5 min before surgery; Topical, 1 g, intraoperatively; 1 g, 3 h, 6 h after surgery	NA
Wang 2017	Intravenously, 1 g, 15 min before surgery; Topical, 1 g, intraoperatively	Rivaroxaban (10 mg/day)
Wang 2019	Intravenously, 1 g, 15 min before surgery; Topical, 1 g, intraoperatively; Intravenously, 1 g, 3 h after surgery	Enoxaparine 4000 IU
Xie 2017	Intravenously, 20 mg/kg, 10 min before surgery; Topical, 60 ml, intraoperatively	Enoxaparine 4000 IU
Xue 2018	Intravenously, 1 kg, 30 min before surgery	Rivaroxaban (10 mg/day)
Yu 2017	Topical, 1 g, intraoperatively	Rivaroxaban (10 mg/day)
Zak 2021	Intravenously, two dose of 1 g, before surgery and during wound closure	NA
Zeng 2021	Intravenously, 1 kg, before surgery	Rivaroxaban (10 mg/day)
Zhou 2017	Intravenously, 1 g, at the initiation of the surgery and just before closure	Rivaroxaban (10 mg/day)

The detailed information of TXA and thromboprophylaxis drugs of two groups. h, hour; min, minute; IU, international unit; kg, kilogram; g, gram; mg, milligram; ml, milliliter

**Fig. 2:**
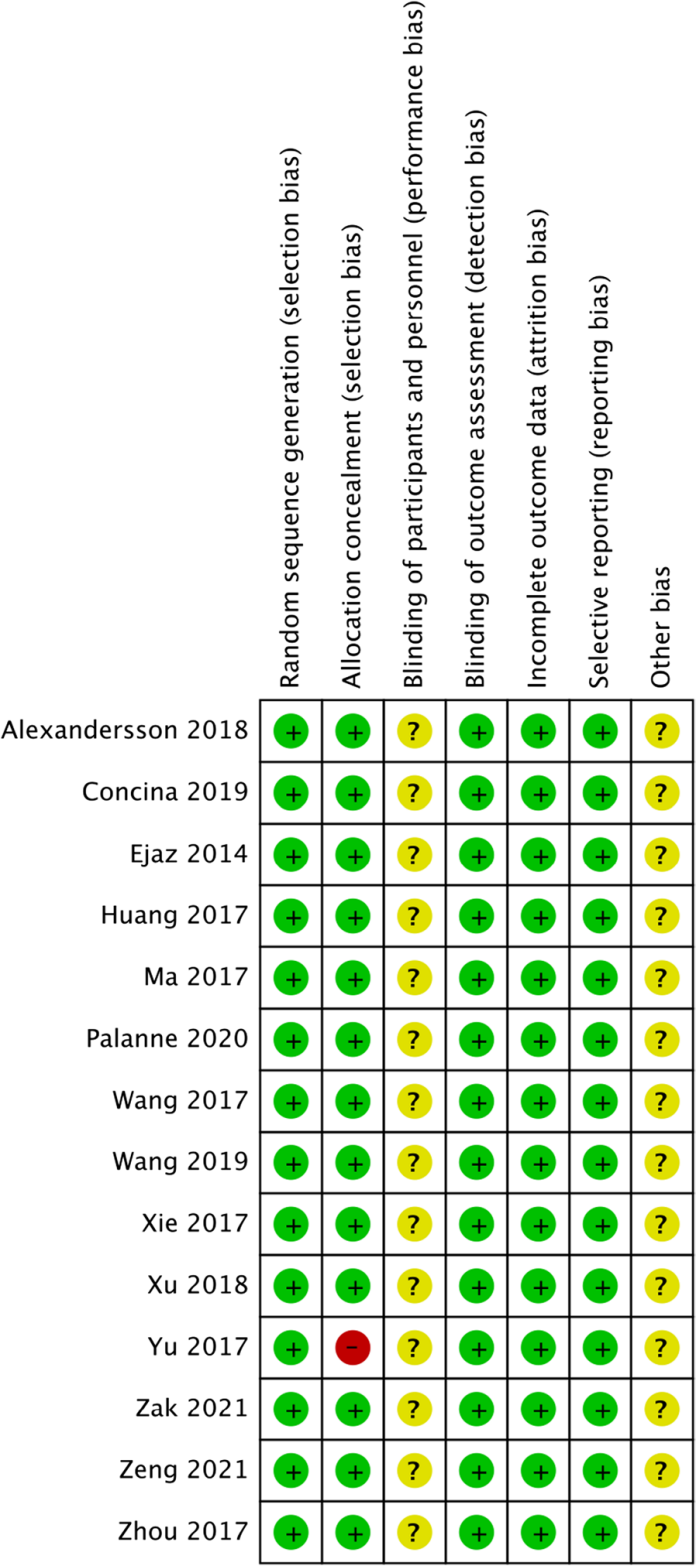
Risk of bias summary: + :no bias; − : bias; ?: bias unknown. Fourteen studies adequately described the correct randomization. Thirteen studies demonstrated sufficient allocation concealment. Four studies described the blinding of participants and personnel. No studies described the blinding of outcome assessment. All thirteen articles retained complete outcome data and avoided selective reporting. We rated as unclear risk of other bias because we can't ignore other potential dangers of biases. As a result, there is low or moderate risk of bias in most of the articles reviewed

**Fig. 3:**
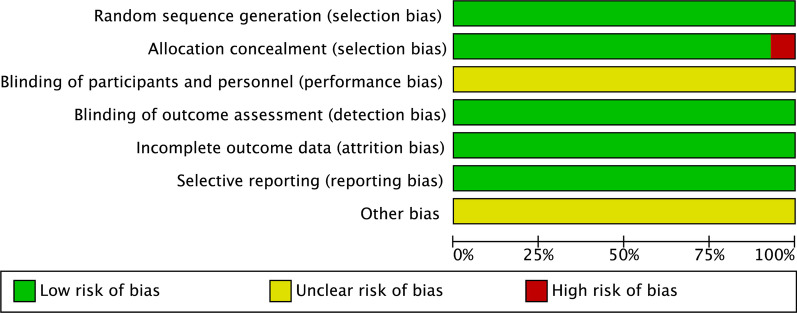
Risk of bias graph. The overall quality of the included studies was considered adequate

The risk of bias summary and bias graph for RCTs is shown in Figs. 2 and 3. Fourteen studies adequately described the correct randomization. Thirteen studies demonstrated sufficient allocation concealment. Four studies described the blinding of participants and personnel. No studies described the blinding of outcome assessment. All thirteen articles retained complete outcome data and avoided selective reporting. We rated as unclear risk of other bias because we cannot ignore other potential dangers of biases. As a result, there is low or moderate risk of bias in most of the articles reviewed (Fig. 2).

### Blood loss

**Fig. 4:**
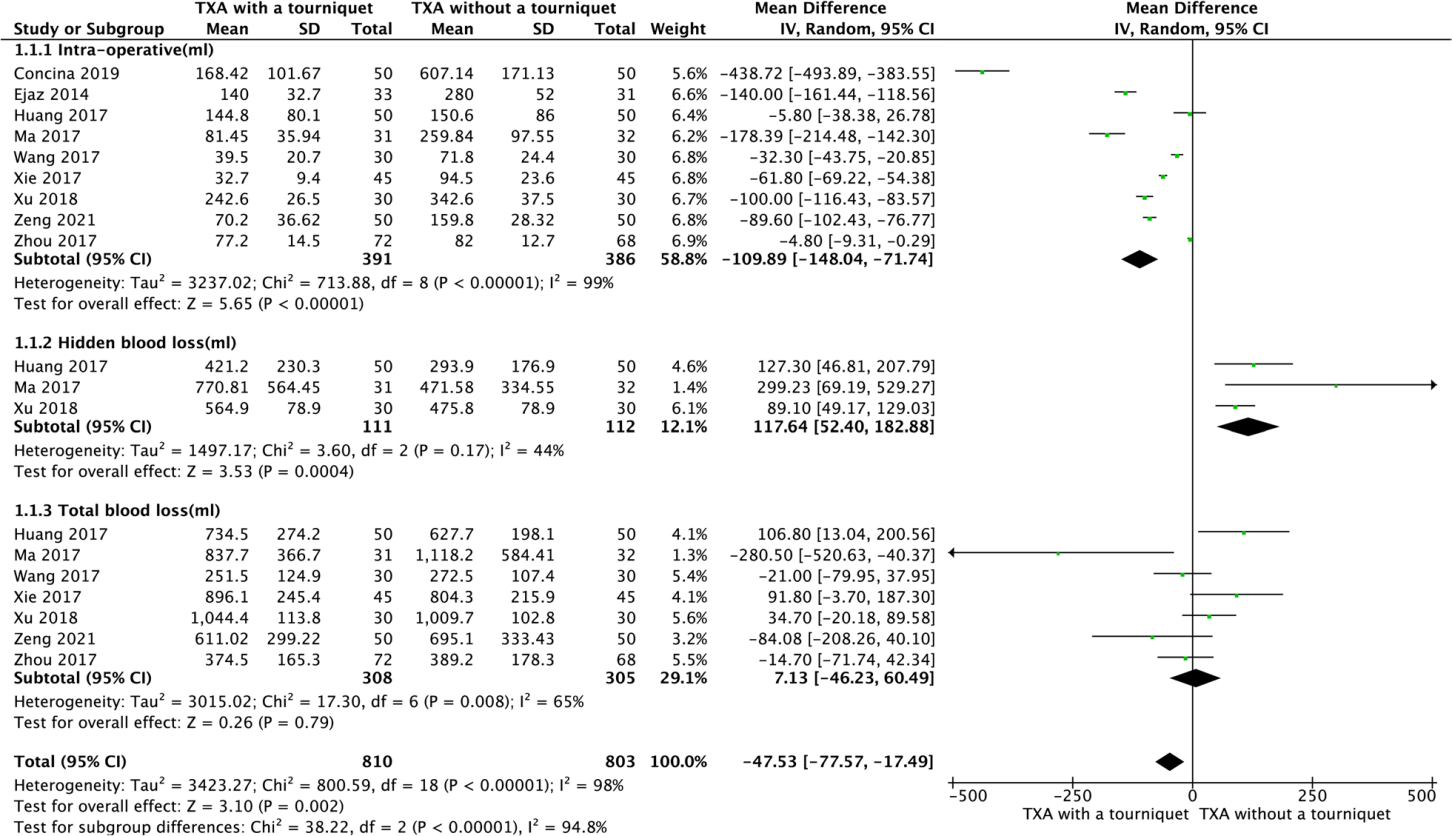
A forest plot diagram showing blood loss. Nine RCTs reported IBL; three RCTs reported HBS and seven RCTs reported total blood loss. The pooled data showed that the TXA with tourniquet group had significantly decreased IBL (MD =  − 109.89, 95% CI [− 148.04, − 71.74], *P* < 0.00001). However, the TXA without tourniquet group has significantly increased HBL (MD = 117.64, 95% CI [52.4, 182.88], *P* = 0.0004). Both groups experienced similar TBL (MD = 7.13, 95% CI [− 46.23, 60.49], *P* = 0.79)

Nine RCTs reported IBL; three RCTs reported HBS and seven RCTs reported total blood loss. The pooled data showed that the TXA with tourniquet group had significantly decreased IBL (MD =  − 109.89, 95% CI [− 148.04, − 71.74], *P* < 0.00001, Fig. 4). However, the TXA without tourniquet group has significantly increased HBL (MD = 117.64, 95% CI [52.4, 182.88], *P* = 0.0004, Fig. 4). Both groups experienced similar TBL (MD = 7.13, 95% CI [− 46.23, 60.49], *P* = 0.79, Fig. 4).

### Drainage volume

**Fig. 5:**
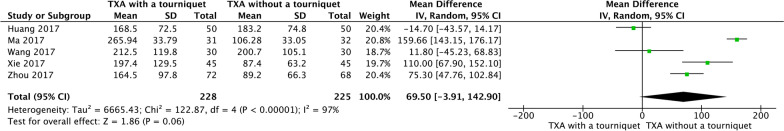
A forest plot diagram showing drainage volume. Five RCTs reported drainage volume. The forest plot showed that the drainage volume was not significantly different between the two groups (MD = 69.50, 95% CI [− 3.91, 142.9], P = 0.06)

Five RCTs reported drainage volume. The forest plot showed that the drainage volume was not significantly different between the two groups (MD = 69.50, 95% CI [− 3.91, 142.9], *P* = 0.06, Fig. 5).

### Decrease in hemoglobin

**Fig. 6:**
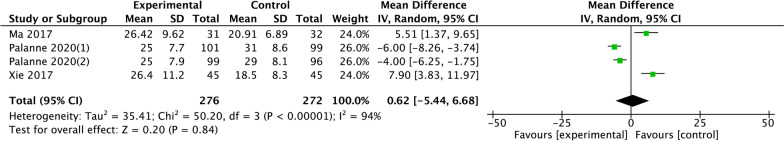
A forest plot diagram showing decrease in hemoglobin. Four RCTs reported a decrease in hemoglobin. The pooled data revealed that the reduction in hemoglobin was not significantly different between the two groups (MD = 7.90, 95% CI [− 5.44, 6.68], *P* = 0.84)

Four RCTs reported a decrease in hemoglobin. The pooled data revealed that the reduction in hemoglobin was not significantly different between the two groups (MD = 7.90, 95% CI [− 5.44, 6.68], *P* = 0.84, Fig. 6).

### Transfusion rate

**Fig. 7:**
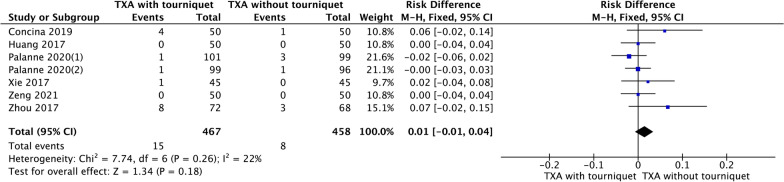
A forest plot diagram showing transfusion rate. Seven RCTs reported the transfusion rate. The forest plot revealed that the transfusion rate was not significantly different between the two groups (RD = 0.07, 95% CI [− 0.02, 0.04], *P* = 0.18)

Seven RCTs reported the transfusion rate. The forest plot revealed that the transfusion rate was not significantly different between the two groups (RD = 0.07, 95% CI [− 0.02, 0.04], *P* = 0.18, Fig. 7).

### Duration of surgery

**Fig. 8:**
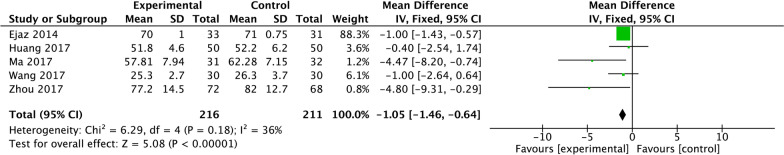
A forest plot diagram showing time of surgery. Five RCT reported duration of surgery, TXA with tourniquet group have significantly decreased time of surgery compared with TXA-NT group (MD =  − 1.05, 95% CI [− 1.46, − 0.64], *P* =  < 0.00001)

Five RCTs reported duration of surgery, TXA with tourniquet group have significantly decreased time of surgery compared with TXA-NT group (MD =  − 1.05, 95% CI [− 1.46, − 0.64], *P* =  < 0.00001, Fig. 8).

### VAS

**Fig. 9:**
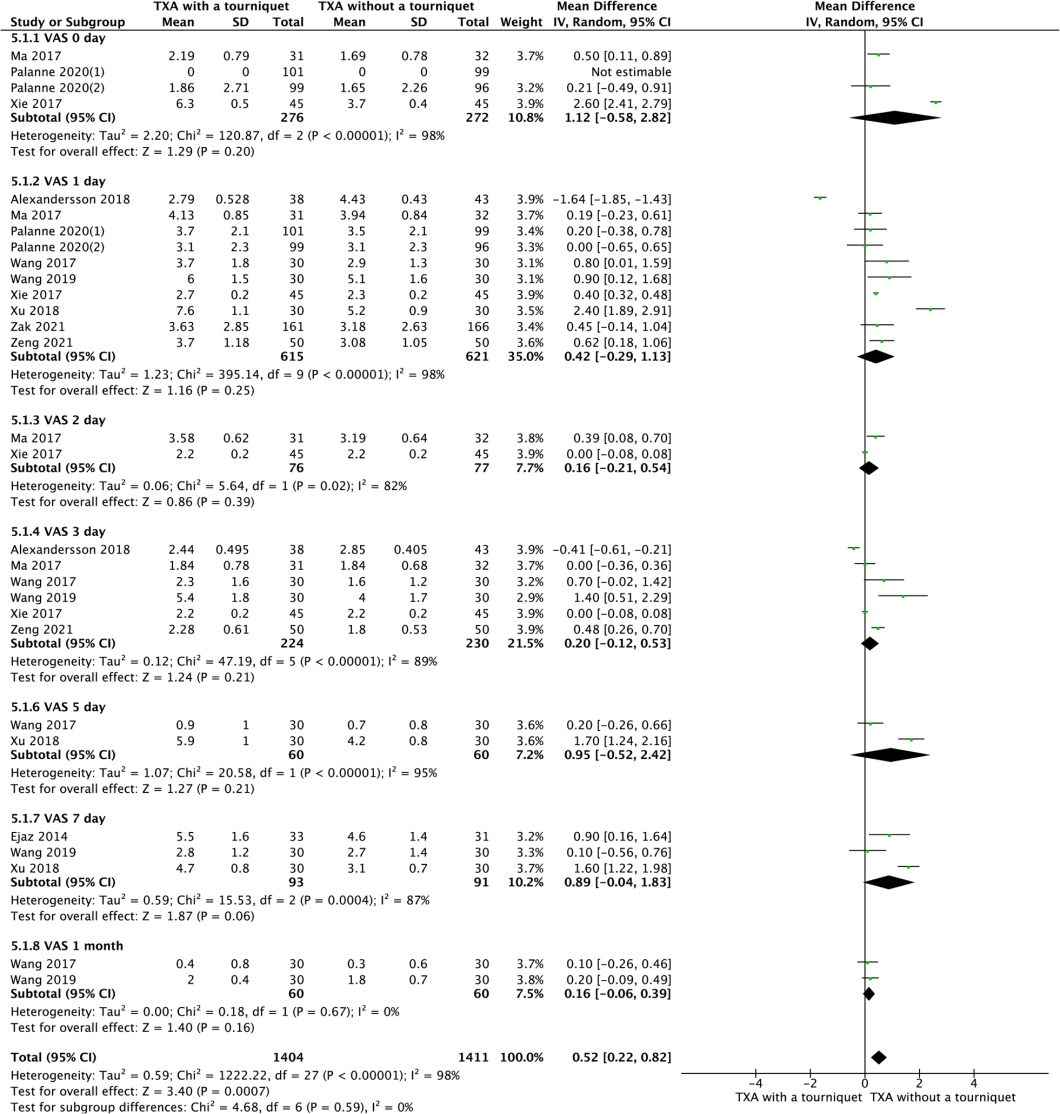
A forest plot diagram showing VAS. Four RCTs reported VAS on the day of surgery. Ten RCTs reported VAS on the first day after surgery. Six RCTs reported VAS on the third day after surgery. Two RCTs reported VAS on the second and fifth day after surgery. Three RCTs reported VAS on the seventh day after surgery. Two RCTs reported VAS at 1 month after surgery. The results of random-effects meta-analysis showed no significant differences between the two groups in the postoperative VAS score at either the day of surgery (MD = 1.56, 95% CI [− 5.0, 3.62], *P* = 0.20), first day (MD = 0.42, 95% CI [− 0.29, 1.13], *P* = 0.25), second day (MD = 0.16, 95% CI [− 0.21, 0.54], *P* = 0.39), third day (MD = 0.20, 95% CI [− 0.12, 0.53], *P* = 0.21), fifth day (MD = 0.95, 95% CI [− 0.52, 2.42], *P* = 0.21), seventh day (MD = 0.89, 95% CI [− 0.04, 1.83], *P* = 0.06)or 1 month after surgery (MD = 0.16, 95% CI [− 0.06, 0.39], *P* = 0.16)

Four RCTs reported VAS on the day of surgery. Ten RCTs reported VAS on the first day after surgery. Six RCTs reported VAS on the third day after surgery. Two RCTs reported VAS on the second and fifth day after surgery. Three RCTs reported VAS on the seventh day after surgery. Two RCTs reported VAS at 1 month after surgery. The results of random-effects meta-analysis showed no significant differences between the two groups in the postoperative VAS score at either the day of surgery (MD = 1.56, 95% CI [− 5.0, 3.62], *P* = 0.20, Fig. 9), first day (MD = 0.42, 95% CI [− 0.29, 1.13], *P* = 0.25, Fig. 9), second day (MD = 0.16, 95% CI [− 0.21, 0.54], *P* = 0.39, Fig. 9), third day (MD = 0.20, 95% CI [− 0.12, 0.53], *P* = 0.21, Fig. 9), fifth day (MD = 0.95, 95% CI [− 0.52, 2.42], *P* = 0.21, Fig. 9), seventh day (MD = 0.89, 95% CI [− 0.04, 1.83], *P* = 0.06, Fig. 9)or 1 month after surgery (MD = 0.16, 95% CI [− 0.06, 0.39], *P* = 0.16, Fig. 9).

### HSS

**Fig. 10:**
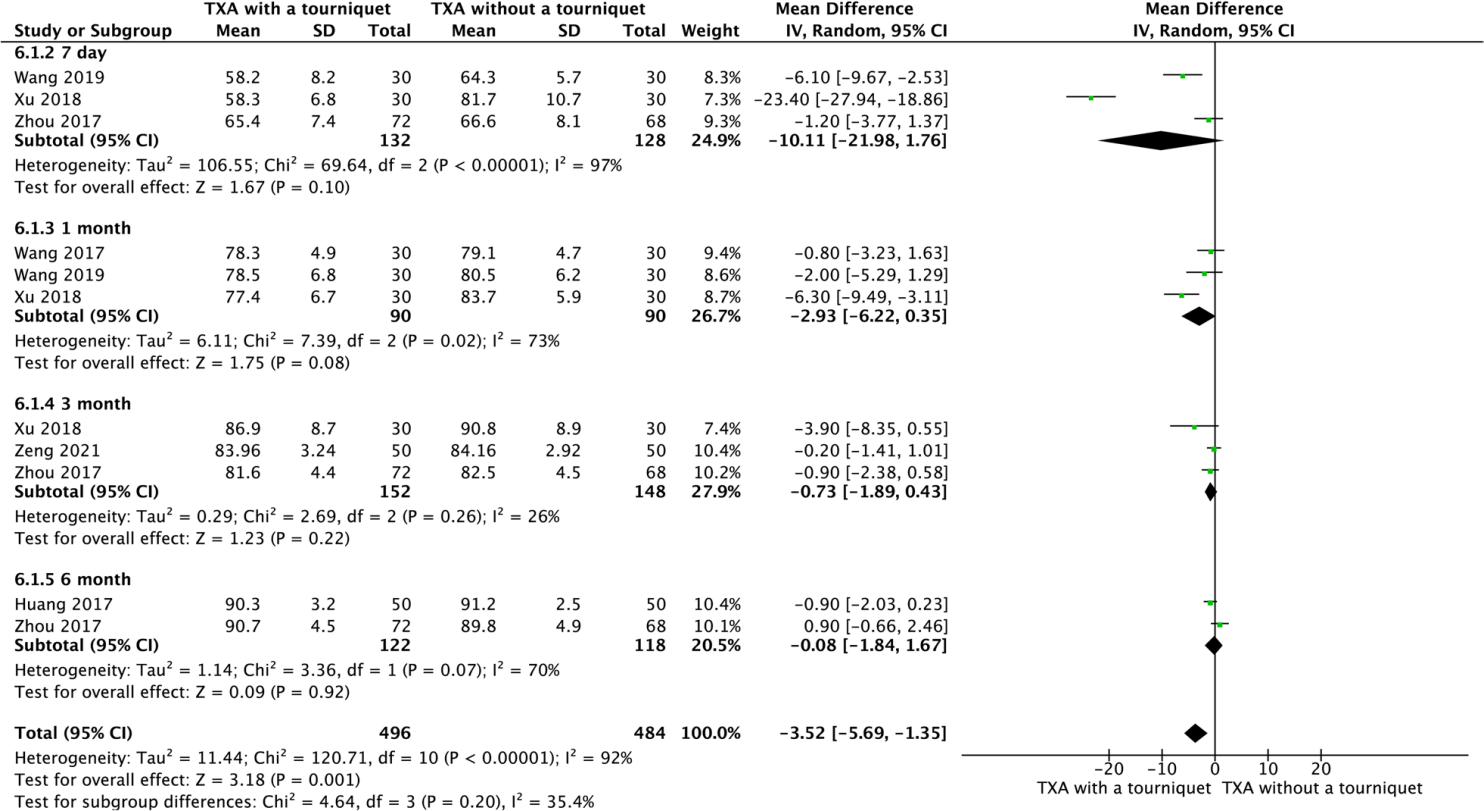
A forest plot diagram showing HSS. Three RCTs reported HSS 7 day, 1 month, 3 month after surgery. Two RCTs reported HSS 6 month after surgery. The pooled results showed that both groups experienced similar HSS scores at either 7 day (MD =  − 10.11, 95% CI [− 21.98, 1.76], *P* = 0.10), 1 month (MD =  − 2.93, 95% CI [− 6.22, 0.35], *P* = 0.08), 3 month (MD =  − 0.73, 95% CI [− 1.89, 0.43], *P* = 0.22) or 6 month after the surgery (MD =  − 0.08, 95% CI [− 1.84, 1.67], *P* = 0.92)

Three RCTs reported HSS 7 day, 1 month, 3 month after surgery. Two RCTs reported HSS 6 month after surgery. The pooled results showed that both groups experienced similar HSS scores at either 7 day (MD =  − 10.11, 95% CI [− 21.98, 1.76], *P* = 0.10; Fig. 10), 1 month (MD =  − 2.93, 95% CI [− 6.22, 0.35], *P* = 0.08; Fig. 10), 3 month (MD =  − 0.73, 95% CI [− 1.89, 0.43], *P* = 0.22; Fig. 10) or 6 month after the surgery (MD =  − 0.08, 95% CI [− 1.84, 1.67], *P* = 0.92; Fig. 10).

### Knee circumference

**Fig. 11:**
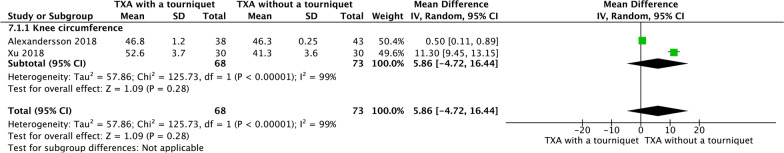
A forest plot diagram showing knee circumference. Two RCTs reported knee circumference. We detected a similar knee circumference between two groups (MD = 5.86, 95% CI − 4.72, 16.44], *P* = 0.28)

Two RCTs reported knee circumference. We detected a similar knee circumference between two groups (MD = 5.86, 95% CI − 4.72, 16.44], *P* = 0.28; Fig. 11).

### Knee ROM

**Fig. 12:**
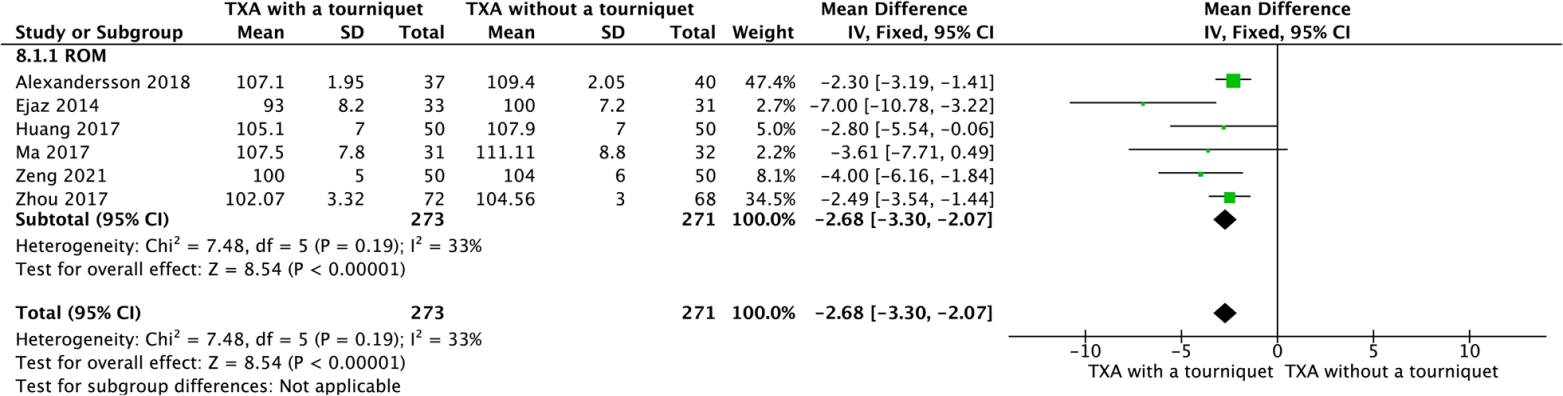
A forest plot diagram showing knee ROM. Six RCTs reported knee ROM. TXA with tourniquet group has significantly decreased knee ROM compared with TXA-NT group (MD =  − 2.68, 95% CI − 3.30, − 2.07], *P* < 0.00001)

Six RCTs reported knee ROM. TXA with tourniquet group has significantly decreased knee ROM compared with TXA-NT group (MD =  − 2.68, 95% CI − 3.30, − 2.07], *P* < 0.00001; Fig. 12).

### LOH

**Fig. 13:**
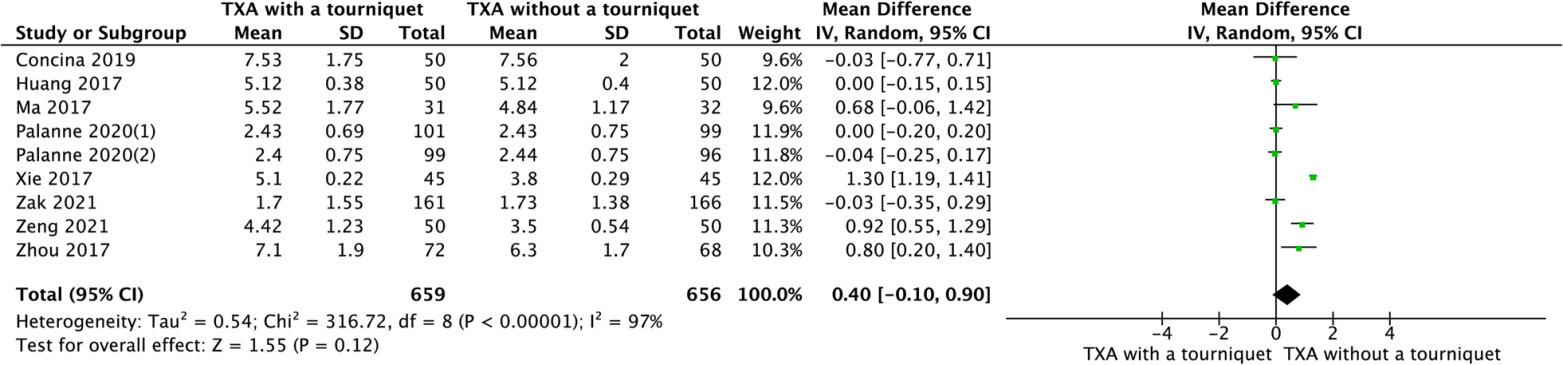
A forest plot diagram showing LOH. Nine RCTs reported LOH. No significant difference was found for LOH between both groups (MD = 0.40, 95% CI − 0.1, − 0.9], *P* = 0.12)

Nine RCTs reported LOH. No significant difference was found for LOH between both groups (MD = 0.40, 95% CI − 0.1, − 0.9], *P* = 0.12; Fig. 13).

### Complications

**Fig. 14:**
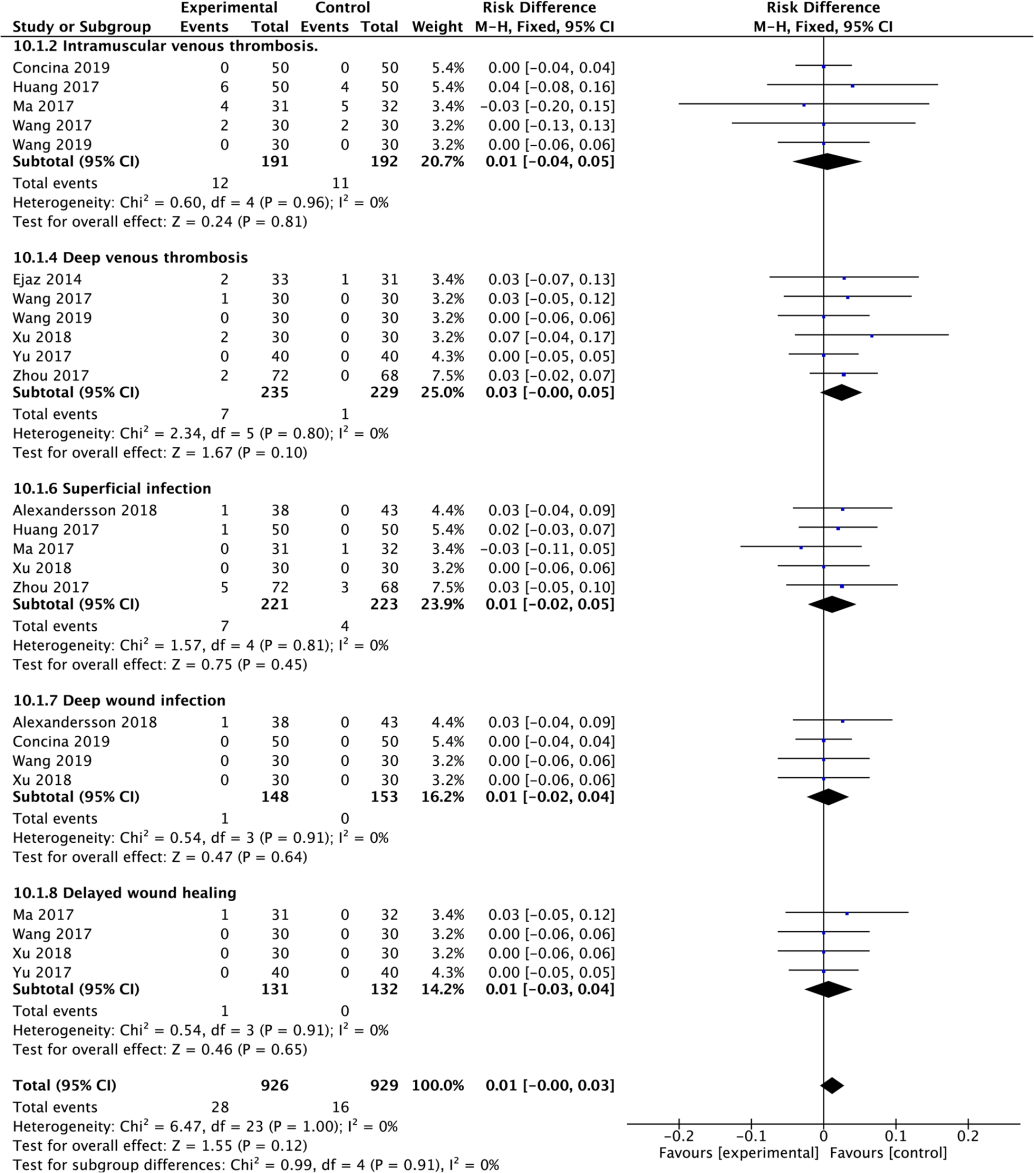
A forest plot diagram showing complications. Five RCTs reported intramuscular venous thrombosis. Six RCTs reported Deep venous thrombosis, five RCTs reported superficial infection, and four RCTs reported deep wound infection. Four RCTs reported delayed wound healing. We detected no significantly difference in terms of intramuscular venous thrombosis (RD = 0.01, 95% CI − 0.04, 0.05], *P* = 0.81), deep venous thrombosis (RD = 0.03, 95% CI − 0.00, 0.05], *P* = 0.10), superficial infection (RD = 0.01, 95% CI − 0.02, 0.05], *P* = 0.45), deep wound infection (RD = 0.01, 95% CI − 0.02, 0.04], *P* = 0.64), delayed wound healing (RD = 0.01, 95% CI − 0.03, 0.04], *P* = 0.65) between two groups

Five RCTs reported intramuscular venous thrombosis. Six RCTs reported deep venous thrombosis, five RCTs reported superficial infection, and four RCTs reported deep wound infection. Four RCTs reported delayed wound healing. We detected no significantly difference in terms of intramuscular venous thrombosis (RD = 0.01, 95% CI − 0.04, 0.05], *P* = 0.81; Fig. 14), deep venous thrombosis (RD = 0.03, 95% CI − 0.00, 0.05], *P* = 0.10; Fig. 14), superficial infection (RD = 0.01, 95% CI − 0.02, 0.05], *P* = 0.45; Fig. 14), deep wound infection (RD = 0.01, 95% CI − 0.02, 0.04], *P* = 0.64; Fig. 14), delayed wound healing (RD = 0.01, 95% CI − 0.03, 0.04], *P* = 0.65; Fig. 14) between two groups.

## Discussion

Our study is the first meta-analysis to identify relevant randomized controlled trials involving TXA plus a tourniquet and use of TXA plus no tourniquet during TKA. This meta-analysis of 15 RCTs that evaluated a total of 1720 TKAs shows that TXA plus tourniquet group can decrease intraoperative blood loss and surgery duration, however increase hidden blood loss and decrease the knee ROM. Our findings suggested that there were no significant differences in terms of total blood loss, decrease in hemoglobin, transfusion rate, drainage volume, VAS, HSS, knee circumference, knee ROM, LOH, and complications between the two groups.

The result showing that the use of a tourniquet plus TXA effectively reduced intraoperative blood loss was consistent with the outcome of previous meta-analysis [35–37]. However, we found the TXA-T group has more hidden blood loss. An explanation for these conflicting results of IBL and HBL indicates that hidden blood loss plays a key role. Tourniquet release can result in ongoing bleeding from cut cancellous bone [38], blood extravasated into the knee joint and adjacent soft tissues [39], or blood loss from hemolysis [40] because of tourniquet-induced ischemia [41, 42]. Furthermore, there are no differences in drainage volume and total blood loss between the two groups, which is inconsistent with the previous meta-analysis. At an earlier meta-analysis [13, 37, 43], they found total blood loss to be significantly lower with a tourniquet. We think the reason for the difference between our study and previous meta- analysis [13, 37, 43] is the TXA used in all RCT studies included in our meta-analysis.

Hemoglobin level and transfusion rate have been recognized as the most objective indicators of actual blood loss. The decrease in hemoglobin and transfusion rate was similar in the TXA-NT group compared with the TXA-T group in our study. Blood transfusion is associated with adverse effects, including hemolytic reactions, infections, morbidity, immunologically mediated diseases, and cost [44]. The result of similar transfusion rate in both groups is consistent with Cai’s recent meta-analysis [45]. They found no significant difference between the tourniquet group and the non-tourniquet group.

A tourniquet will provide surgeons with a bloodless surgery field to facilitate the clear identification of anatomical structures with less electrocoagulation and wound irrigation during surgery, which might help shorten the operation time. Our result showed tourniquet with TXA use reduced surgery duration, which was consistent with previous studies [2, 35, 38]. So a reduction of course of surgery is a potential benefit of tourniquet use with TXA in TKA.

Pain relief in the early postoperative period after TKA is crucial in facilitating early recovery. Whether the use of tourniquets will increase postoperative pain remains controversial. Theoretically, tourniquet use may increase thigh pain and swell due to lower limb blood flow occlusion and ischemia–reperfusion injury. Our study identified no difference in pain intensity at either the day of surgery, first day, second day, the third day, fifth day, the seventh day, or 1 month after surgery. Although tourniquet pressure, time, and time of postoperative pain evaluation were variable across studies, we found that these factors of all included RCTs were comparable between experimental and control groups, so endpoints like VAS, ROM, and LOS could still be properly assessed. We also have tried our best to evaluate VAS based on time points. Our results of VAS were inconsistent with previous studies. [25, 46, 47]. It may be related to the tourniquet pressure in our tourniquet group. In our study, lower or personalized tourniquet pressure was used in 5 of the 11 RCTs. Worland et al. [48] showed an essential correlation between higher tourniquet pressure and more thigh pain in the immediate postoperative period.

Knee flexion ROM is often used to evaluate short-term effectiveness. Besides, discharge from the hospital is dependent on the mobility of patients following TKA. We found significantly decreased knee ROM in TXA-T group compared with TXA-NT group, which is consistent with the previous systematic reviews [35, 49]. We think the possible reasons are as follows: (1) using a tourniquet could injure the nerve and the skeletal muscle, even causing rhabdomyolysis [49]; (2) the tourniquet might cause reperfusion injury, which might cause a degree of muscle fibrosis; (3) there was some delay in the nerve conduction and electromyography changes of the extensor apparatus when using tourniquet [49]. No significant difference was also found in terms of knee circumference between the two groups. These findings seem logical, given that we found no significant difference in terms of VAS.

The analysis of the postoperative HSS at either 7 days, 1-month, 3 months or 6 months after the surgery also did not reveal a difference. HSS might be affected by many factors such as pain, ROM, function, muscle force, and flexion deformity. Moreover, the effect of a tourniquet application plus TXA on HSS needs to be further confirmed by more high-quality studies.

As for complications, we observed no significant difference in terms of IMVT, DVT, superficial infection, deep wound infection, delayed wound healing between the two groups. Although TXA use in TKA didn’t increase thromboembolic events [50–53], perhaps one of the more significant clinical concerns regarding tourniquet use plus TXA is its association with thromboembolism. No significant difference was found between groups regarding the rate of intramuscular venous thrombosis and deep venous thrombosis in our study. Several studies have investigated the incidence of venous thrombosis with the use of the tourniquet [3, 13, 14, 36, 54]. However, the evidence is mixed because of heterogeneous study groups and designs, making it difficult to compare. Nonetheless, we cannot underscore the importance of chemoprophylaxis following TKA regardless of tourniquet use. DVT was detected in 81% of patients when all the patents only received mechanical compression but no chemoprophylaxis following TKA of tourniquet use [55].

The current meta-analysis has several limitations: First, there is a high heterogeneity of blood loss caused by the different methods for measuring blood loss, separate application of a tourniquet, different operative techniques, and different perioperative management as the drain and anticoagulant therapy. The reliability of results may be influenced by this heterogeneity. Second, the studies' comparability was complicated through the different measurement methods and follow-up examination time points; however, we have tried our best to evaluate results based on time points. Third, the tourniquet time, the time for loosening the tourniquet, and the cuff pressure used were also not uniform. Fourth, there are no worldwide uniform guidelines for performing total knee arthroplasty. Different surgical techniques (such as the selection of approach, anesthesia methods, patellar resurfacing, and type of prosthesis) were used in the individual studies. Fifth, the tourniquet may have impact on patella tracking; however, there were too few studies comparing patella tracking between two groups, so we did not have further research on the patella tracking in our study.

## Conclusion

No big differences could be found by using or not tourniquet with TXA. Some benefits are related to operation time and less intra-operative blood loss by using tourniquet and TXA; however, using the tourniquet and TXA was also related to more hidden blood loss and less knee range of motion. These are obvious conclusions that are confirmed after this meta-analysis. Given our meta-analysis' relevant possible biases, we required more adequately powered and better-designed RCT studies with long-term follow-up to reach a firmer conclusion.

## Data Availability

The datasets generated and analyzed during the current study are available from the corresponding author on reasonable request.

## Abbreviations

Cis: Confidence intervals
RCTs: Randomized controlled trials
RR: Risk ratio
OR: Odds ratio
VMD: Weighted mean difference
TXA: Tranexamic acid
TKA: Total knee arthroplasty
OA: Osteoarthritis
RA: Rheumatoid arthritis
BMI: Body mass index
VAS: Visual Analogue Scale
HSS: Hospital for Special Surgery
ROM: Range of motion
IBL: Intra-operative blood loss
HBL: Hidden blood loss
TBL: Total blood loss
LOH: Length of stay
IMVT: Intramuscular venous thrombosis
DVT: Deep venous thrombosis
